# Evaluation of biases present in the cohort multiple randomised controlled trial design: a simulation study

**DOI:** 10.1186/s12874-017-0295-7

**Published:** 2017-01-31

**Authors:** Jane Candlish, Alexander Pate, Matthew Sperrin, Tjeerd van Staa, on behalf of GetReal Work Package 2

**Affiliations:** 1 Health eResearch Centre, Farr Institute for Health Informatics Research, University of Manchester, Vaughan House, Portsmouth Road, Manchester, M13 9PL UK; 2 School of Health and Related Research, University of Sheffield, 30 Regent St, Sheffield, S1 4DA UK; 3 Utrecht Institute for Pharmaceutical Sciences, Utrecht University, Utrecht, The Netherlands

**Keywords:** Trials within cohorts, Cohort multiple randomised controlled trial, Pragmatic trial, Instrumental variable

## Abstract

**Background:**

The cohort multiple randomised controlled trial (cmRCT) design provides an opportunity to incorporate the benefits of randomisation within clinical practice; thus reducing costs, integrating electronic healthcare records, and improving external validity. This study aims to address a key concern of the cmRCT design: refusal to treatment is only present in the intervention arm, and this may lead to bias and reduce statistical power.

**Methods:**

We used simulation studies to assess the effect of this refusal, both random and related to event risk, on bias of the effect estimator and statistical power. A series of simulations were undertaken that represent a cmRCT trial with time-to-event endpoint. Intention-to-treat (ITT), per protocol (PP), and instrumental variable (IV) analysis methods, two stage predictor substitution and two stage residual inclusion, were compared for various refusal scenarios.

**Results:**

We found the IV methods provide a less biased estimator for the causal effect when refusal is present in the intervention arm, with the two stage residual inclusion method performing best with regards to minimum bias and sufficient power. We demonstrate that sample sizes should be adapted based on expected and actual refusal rates in order to be sufficiently powered for IV analysis.

**Conclusion:**

We recommend running both an IV and ITT analyses in an individually randomised cmRCT as it is expected that the effect size of interest, or the effect we would observe in clinical practice, would lie somewhere between that estimated with ITT and IV analyses. The optimum (in terms of bias and power) instrumental variable method was the two stage residual inclusion method. We recommend using adaptive power calculations, updating them as refusal rates are collected in the trial recruitment phase in order to be sufficiently powered for IV analysis.

## Background

Clinical trials are facing increasing costs, complexities, and poor accrual, all threatening the viability of the current clinical trial model [1]. There is a vital need for trial designs that promote increased external validity and cost efficiency so we can continue to deliver medical benefits to patients [2]. To this end, there have been frequent calls for more pragmatic generalizable trials [3, 4]. A simplification of trial eligibility criteria and integration of routine electronic health records to monitor trial outcomes should play a key role in the development of pragmatic trials [1, 3]. The cohort multiple randomised controlled trial design [5] (cmRCT, also referred to as trial within cohorts design) is a simple and efficient pragmatic trial design that could address some of the issues discussed. In a cmRCT design we identify a large observational cohort, and at the outset consent is obtained from participants both for random allocation to interventions in the future, and to use their medical records data. For a given intervention, eligible patients are identified from the cohort and a random selection are offered the intervention. The remaining eligible patients are assigned to the control group and are not formally recruited into the trial, thus addressing some of the issues with trial accrual.

The cmRCT design offers several advantages. First, the use of a large observational cohort enables multiple trials over time and as patients all come from the same cohort comparability of these trials is improved [5]. Second, electronic healthcare records reduce recruitment and follow-up costs [6], making it possible to have a large control arm requiring minimal additional costs for follow-up. Third, both the intervention and standard of care are delivered at the point of care enabling us to assess the effectiveness of both interventions in real life conditions as opposed to under strict trial conditions.

A cmRCT design has several limitations [5, 7]. The use of routine data will require a mix of skill sets from both clinical trial and observational research, possibly increasing costs. Loss to follow-up may be a significant issue if linkage of routine data linkage is not possible. The comparator is standard of care, this may contain a variety of treatments with some self-selected by the patient so is somewhat an unknown. The feasibility of standardised outcome measures may also be challenging when using routine data. Blinding of participants in the intervention is not possible in the cmRCT design. If the cohort of eligible patients is too small in a cmRCT and refusal is high, it is not possible to keep recruiting until the required power is achieved as this will start oversampling from the control arm leading to a less powerful trial than the standard two arm. A notable limitation is that only patients randomly selected to receive the experimental intervention have the option to refuse the allocated treatment. This refusal may differ from the refusal we see in routine care due to the experimental nature of the study. Refusal can potentially lead to bias in the treatment effect estimate and loss of power. Though the cmRCT design is a recent proposal, its uptake has been fairly broad in application [8–12] and ongoing cmRCTs have scarcely considered the matter of refusal. Where mention of intended analysis methods were available, intention-to-treat was always used. Only one trial made explicit assumptions about the proportions of refusers in the intervention arm, assuming a 20% refusal rate and planned to use complier average causal effect (CACE) [13] analysis if refusal in the intervention arm was large [10], another noting CACE analysis as a secondary analysis method [9]. CACE analysis provides an unbiased treatment effect estimate for individuals who comply with the protocol, unlike intention-to-treat or per-protocol; however, there is generally a loss of power.

The effects of non-uptake of the experimental intervention in cmRCT are unclear. In this study, we undertook a simulation study to test the validity of the cmRCT design in the presence of treatment refusal. The objectives were to assess the bias present in the cmRCT design due to differential refusals and any loss in statistical power. We identify scenarios where cmRCT are viable designs for point-of-care trials and suggest alternate statistical methods, such as instrumental variable analysis, which can be used to reduce bias.

Randomised trials are often divided into two types, individually randomised – individuals randomized to trial arm - or cluster randomized trials – clusters (groups of individuals or communities) randomized to trial arm. In certain scenarios cluster trials can be more appropriate (for instance when we anticipate contamination between trial arms), they can also improve accrual and reduce costs. However, cluster trials require specific design, statistical analysis and interpretation and are not always beneficial. In cluster trials we generally anticipate an inflation of the variance due to correlation among individuals in the same cluster, therefore, they require larger sample sizes than individually randomized trials designed to answer the same research question (if an individually randomized trial is possible) and more complex analysis to account for the correlation of outcomes within a cluster. This paper focusses on individually randomised cmRCTs, representative of the randomization used in the original cmRCT design and existing cmRCT studies [5]. The case of a cluster cmRCT has been investigated by Pate et al. [14]; differing effects of the refusal on power were found between the cluster and individual trial scenarios. As sample sizes for trials using the cmRCT design will be limited to the size of the cohort, the benefits and requirements for cluster level randomization should be considered with caution.

## Methods

Data were simulated to represent hypothetical trials using a cmRCT design with randomisation of individual patients. The cohort of patients was those receiving standard of care for a particular condition. The trial assessed the effect of an intervention compared with standard of care, with an outcome of time to cardiovascular disease (CVD) event and effect estimate the hazard ratio. The simulations replicated a cmRCT study which assessed the effect of an intervention compared with standard of care for patients at risk of cardiovascular disease (CVD) and eligible for lipid lowering drugs [14]. The outcome of interest was the time to CVD event. We define treatment effect, the parameter of interest, as the complier average causal effect (defined counterfactually) of the treatment (taken per protocol) on (hazard of) outcome. Refusal of the intervention and correlation between this refusal and baseline risk were varied to create different trial scenarios. It was assumed that the underlying risk for the outcome of interest varied between patients. We used bias (error in the estimation of the causal treatment effect), statistical power, and standard error of the mean treatment effect as measures of performance.

### Analysis methods

We assessed the bias and variance of effect estimators associated with different analysis methods when treatment refusal was present in the intervention arm of a cmRCT study. The methods included: intention-to-treat (ITT), per protocol (PP) and two instrumental variable (IV) analysis techniques. Current trial guidance recommends ITT analysis [15]; however, this dilutes the treatment effect in the presence of refusal which may result in biased estimates of the causal effect of a treatment, according to our specific definition of treatment effect above [16]. PP analysis excludes people from the study who do not follow protocol, generally yielding biased results as adherence to protocols is often non-random [17]. IV analysis estimates the effect of exposure (X) on the outcome (Y), accounting for all unmeasured confounding. The IV technique is a two stage analysis; the first stage assesses how an unbiased instrument (randomisation, Z) predicts the exposure (treatment received, X); the second stage uses this information to understand how the exposure predicts the outcome (Y). The performance measures used to assess the four methods were the bias of the estimate of treatment effect, statistical power and standard error.

**Fig. 1:**
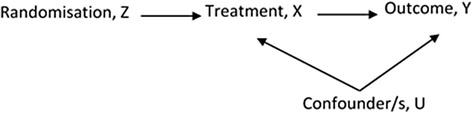
Directed acrylic graph representing randomised trial with treatment refusal

Figure 1 represents the causal relationships required for an IV analysis in a trial. The three main assumptions for the validity of an instrumental variable are: it is strongly associated with exposure, it has no effect on the outcome other than through the exposure, and it is independent of confounders of the exposure outcome relationship [18]. In an RCT randomisation (Z) meets these assumptions; however, the strength of association with exposure depends upon the non-compliance rates. The performance of two IV approaches were compared, the two stage predictor substitution (2SPS) method and the two stage residual inclusion (2SRI) method [19, 20]. The 2SPS method is a straightforward two stage regression approach. First, regress IV on exposure, *Z* on *X*, and let $\widehat{\mathrm{X}}= E\left( X\Big| Z\right)$. In the second stage fit a model for the outcome, *Y*, including $\widehat{\mathrm{X}}$ as a covariate. The coefficient of $\widehat{\mathrm{X}}$ from the second stage model is the IV estimated treatment effect of interest. Similarly, in the first stage of the 2SRI method regress *Z* on *X*, and then calculate the residual term *R* = *X* − *E*(*X*|*Z*). In the second stage, fit a model for the outcome, *Y*, including both treatment received, *X*, and residuals, *R*, as covariates in the model. The coefficient of *X* in the second stage model is the IV estimated treatment effect.

### Simulation overview

Simulations were undertaken using statistical software SAS version 9.4 (SAS Institute Inc, Cary, NC) and plots in R version 3.1.2 followed standard guidelines [21]. Simulations replicated a cmRCT study evaluating the effect of an intervention compared with standard of care for patients at risk of CVD [14]. The outcome was time until CVD event, which is of direct importance to the patient, as opposed to some surrogate endpoints [22]. Simulated patient characteristics included: baseline risk of a CVD event (for example, acute myocardial infarction/stroke (fatal or non-fatal) and death attributable to CVD), time to non-CVD mortality, and probability of refusing the intervention. If non-CVD mortality event occurred before the CVD they were treated as censored. Clinician probability of refusing to offer the intervention was also simulated. The data generating models for time to both CVD event and mortality were Weibull distributions, with distribution parameters chosen to give a correlation of 0.25 between CVD and mortality to represent informative censoring. The distribution parameters were chosen so the mean and standard deviation of the ten year CVD risk were representative of the population eligible for lipid lowering drugs, 21.1 and 8.7% respectively [14]. Refusal was only present in the intervention arm. We assume the treatment has no effect on the competing risk of death from other causes than CVD event.

We used Weibull baseline hazard functions for all individuals for time until CVD event, *T*_*c*_, and time until mortality, *T*_*m*_. Individual risks were created by incorporating individual random effects to the Weibull baseline hazard function. Given that the shape parameter of a Weibull distribution is fixed, then the proportional hazards assumption holds. The intervention effect used was an average reduction in ten year CVD risk by 25%. The trial follow-up used, *T* ^*max*^, was three years as a plausible follow-up period in a survival trial. All simulation variables are presented in Table 1.

**Table 1:** Variables included in the simulation study, generating models, and notation

Variable description	Generating models and notation
Number of simulated data sets	*N*_*sim*_
Sample size each data set	*N*_*obs*_
Treatment allocated	*Z*_*i*_ = 0/1 for standard of care/intervention
Treatment received	*X*_*i*_ = 0/1 for standard of care/intervention
Baseline hazard function for time until CVD event	${h}_0\left({t}_c\right)={\gamma}_c{\lambda}_c^{\gamma_c}{t}_c^{\gamma_c-1},\kern0.5em {\lambda}_c=36,\kern0.62em {\gamma}_c=1.2$
Baseline hazard function for time mortality	${h}_0\left({t}_m\right)={\gamma}_m{\lambda}_m^{\gamma_m},\kern0.5em {\lambda}_m=55,\kern0.5em {\gamma}_c=1.2$
Individual random effects	*ε*_*i*_ ~ *N*(0, *σ* ^2^), *σ* = 0.7
Intervention effect	*β* = − 0.32
Individual hazard function for time until CVD event	$h\left({t}_{i c}\right) = {h}_0\left({t}_c\right)\ {e}^{\left(\beta {X}_i+{\varepsilon}_i\right)}$
Individual hazard function for time until mortality	$h\ \left({t}_{i m}\right)={h}_0\left({t}_m\right)\ {e}^{\left({\varepsilon}_i\right)}$
Probability of a patient refusing intervention if offered and average patient refusal	$$ \begin{array}{l}{p}_i, p={\varSigma}_i{p}_i/{N}_{obs}\\ {}\end{array} $$
Probability of clinician refusing to offer the intervention to the patient and average clinician refusal	$$ \begin{array}{l}{q}_i, q={\varSigma}_i{q}_i/{N}_{obs}\\ {}\end{array} $$
Trial follow-up time in years	*T* ^*max*^ = 3
Censoring indicator	*C*_*i*_ = *I*(*T*_*ic*_ ≥ min(*T*_*im*_, *T*_*max*_ ))
Observed outcome	*Y*_*i*_ = min(*T*_*ic*_, *T*_*im*_, *T*_*max*_)
Observed trial data	{*Y*_*i*_, *C*_*i*_, *Z*_*i*_, *X*_*i*_}

Different trial scenarios were generated through varying both the average refusal probability and the correlation of refusal with individual risk. The average patient refusal probability, *p*, and average clinician refusal probability, *q*, took values of 0.1, 0.2, or, 0.3. Different correlation scenarios were created by defining an upper limit (UL) and lower limit (LL) that all individuals’ refusal probability lies between and assigning refusal probabilities equally spaced between these values. Patients were ordered (ascending or descending) by their individual random effect *ε*_*i*_. Correlation between CVD risk and refusal probability took values defined as zero, low, medium, or high using upper and lower limits for individual refusal probabilities $\left( LL, UL\right)\in \Big\{\left( p, p\right),\left(\frac{2 p}{3},\frac{4 p}{3}\right),\left(\frac{p}{3},\frac{5 p}{3}\right),\left(0,2 p\right)$. For each scenario we simulated 1000 realisations of the trial data.

Sample sizes were calculated under two different recruitment methods: recruitment without refusal assumed no refusal and recruitment with refusal used the expected refusal rate in the sample size calculation (this assumes refusal is non-informative for the sample size calculation – not for estimating effects). Both recruitment methods were used for all simulated trial scenarios. We calculated the required sample size using bootstrap methods [23], an unequal randomisation method of 4:1 standard of care:intervention, 0.80 statistical power, a type I error rate of 0.05 and a minimum treatment effect tested of an average reduction in ten year CVD risk by 25% (taken as the minimum clinically important effect). If a trial budget is fixed but there is no limitation to the total sample size then an increased statistical power can be obtained by sampling more patients from the control arm [24], hence the unequal randomisation ratio. Cox proportional hazard survival models were used to provide effect estimates under each analysis method.

### Simulation steps

We simulated 1000 trials each with size *N*_*obs*_, *i* = 1, …, *N*_*obs*_ for each different combination of parameters. For *j* = 1, …, 1000 the following procedure was undertaken to simulate hypothetical time to event trial data:

1. Individuals risks: Generate individual random effects, *ε*_*i*_ s for each patient, *i* = 1, …, *N*_*obs*_.
2. Refusal probabilities: Order patients by their individual random effect, *ε*_*i*_. Generate refusals either randomly or related to underlying CVD risk by relating them with individual random effect order *ε*_*i*_. The probability of a patient refusing intervention if offered it is *p*_*i*_ and the probability of clinician refusing to offer the intervention to the patient is *q*_*i*_.
3. Randomisation: Randomise to standard of care *Z*_*i*_ =0, or intervention, *Z*_*i*_ =1, on a 4:1 basis.
4. Treatment: Generate the treatment actually received, *X*_*i*_ =0/1 for standard of care/intervention. All those in control arm (*Z*_*i*_ = 0) receive standard of care (*X*_*i*_ = 0) and those randomised to intervention arm (*Z*_*i*_ = 1) receive either intervention or standard of care dependent upon their refusal, *X*_*i*_ = min {*Bernoulli*(1 − *p*_*i*_), *Bernoulli*(1 − *q*_*i*_)}.
5. Hazard rate: Apply intervention effect, $\beta =-0.32$, and individual random effects to the Weibull baseline hazard function to give $h\left({t}_{i c}\right) = {h}_0\left({t}_c\right)\ {e}^{\left(\beta {X}_i+{\varepsilon}_i\right)},\mathrm{and}\kern0.5em  h\ \left({t}_{i m}\right)={h}_0\left({t}_m\right)\ {e}^{\left({\varepsilon}_i\right)}.$
6. Time to event: Simulate individual time to event *T*_*ic*_ and *T*_*im*_ from corresponding Weibull distributions.
7. Censoring: Generate *C*_*i*_ the censoring indicator *C*_*i*_ =0/1 if outcome is an observed event/censored. We observe the continuous outcome *Y*_*i*_ = min(*T*_*ic*_, *T*_*im*_, *T*_*max*_) and the observed trial data is then {*Y*_*i*_, *C*_*i*_, *Z*_*i*_, *X*_*i*_}, a set of censored survival data, treatment allocations, and treatments received.
8. Analysis: Using ITT, PP, 2SPS, and 2SRI methods fit a Cox proportional hazards model to estimate the effect estimate ${\widehat{\beta}}_j$ and the corresponding *p*-value, *s*_*j*_ for each of the four different estimators.

The same set of simulated data sets were used to compare different methods for the same scenario; however, for each scenario a different set of data sets was generated [21]. This enables differences between methods to be detected. The mean effect estimate $\overline{\beta}={\displaystyle \sum_j}{\widehat{\beta}}_j/1000$, relative bias $\left(\overline{\beta}-\beta \right)/\beta$, and statistical power I_j_(*p*_*j*_ < 0.05)/1000 were calculated for each scenario where *j* = 1, …, 1000. The empirical standard error of the mean effect estimate﻿, $\overline{\beta},$ over all simulations ﻿was calculated as $S. E.\left(\overline{\beta}\right)= S. D.\left({\widehat{\beta}}_j\right)/1000$.

## Results

**Fig. 2:**
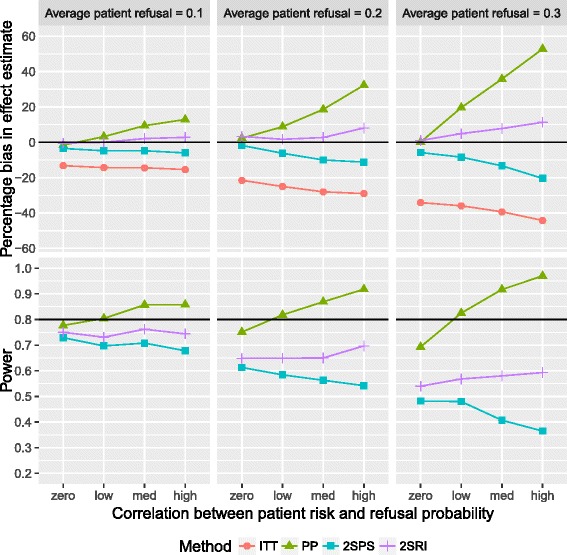
Percentage bias (*top*) and power (*bottom*) against correlation patient risk and refusal probability using recruitment without refusal and positive correlation between refusal and risk: both plots are paneled by the refusal probabilities and present results for all four analysis methods. *Black* reference lines represent empirical power and zero bias

Figure 2 shows the influences of refusal on the causal effect estimate of the treatment (taken per protocol) on hazard of outcome and power (using recruitment without refusal). This was done for a positive correlation between refusal probability and underlying risk for the outcome. PP increasingly overestimates the effect estimate as correlation strengthens, refusal probabilities of 0.1 correlated to risk led to bias of between 3.3 and 12.8%; the corresponding power increased. ITT estimates were biased; average refusal probabilities of 0.2 underestimated the treatment effect by between 21.5 and 29.0%. As the correlation between refusal and risk strengthened the ITT estimate underestimated the effect with increasing magnitude. Both IV methods were less biased than PP or ITT estimates in all scenarios in Fig. 2. An average refusal of 0.2 and high correlation resulted in bias of −11.2% with 2SPS and 8.1% with 2SRI, compared to −29.0% with PP and 32.3% with ITT. The 2SRI method provided the most accurate effect estimate in all scenarios when there was correlation between refusal and risk. The IV and ITT analyses resulted in a lower power than the expected 0.80, the power of 2SRI fluctuated between a 0.65 and 0.70. Statistical power of both 2SPS and ITT were equal and generally decreased as refusal and correlations increased.

**Fig. 3:**
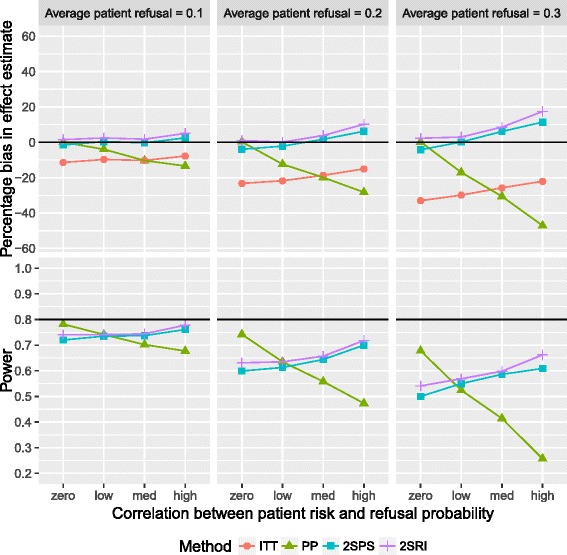
Percentage bias (*top*) and power (*bottom*) against correlation patient risk and refusal probability using recruitment without refusal and negative correlation between refusal and risk: both plots are paneled by the refusal probabilities and present results for all four analysis methods. *Black* reference lines represent empirical power and zero bias

The bias of the effect estimate and statistical power for negative correlations between refusal and event risk are presented in Fig. 3 (using recruitment without refusal). PP analysis provides underestimated effect estimates, increasing as both refusal and correlation increased; the corresponding power decreased. An average refusal of 0.1 negatively correlated with risk yielded an underestimate of the effect estimate of between 4.0 and 13.4%, respective power between 0.78 and 0.68. The ITT analysis again underestimated the effect estimate, however, here with decreasing magnitude as correlations strengthened. Both IV methods followed similar patterns to one another, overestimation and power generally increasing as correlation strengthened. It is the correlation that most affects the IV analyses, with bias only reaching greater than 10% when refusal is 0.3 and correlation is high.

**Fig. 4:**
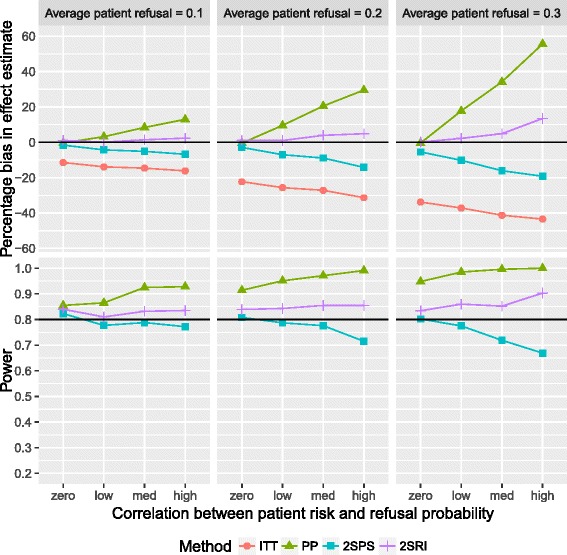
Percentage bias (*top*) and power (*bottom*) against correlation patient risk and refusal probability using recruitment with refusal and positive correlation between refusal and risk: both plots are paneled by the refusal probabilities and present results for all four analysis methods. The *black* reference lines represent empirical power and zero bias

**Fig. 5:**
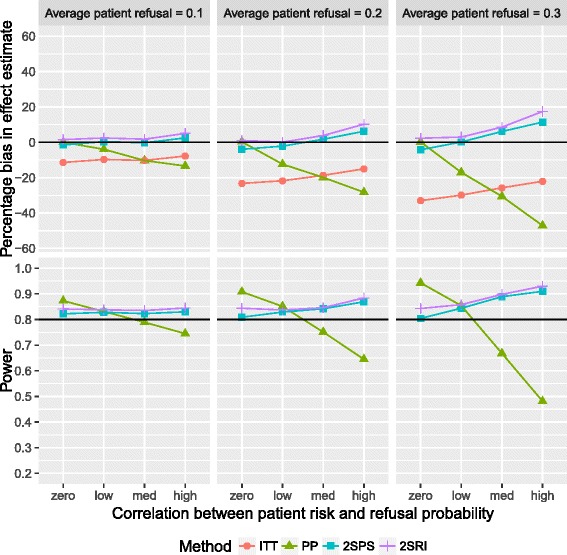
Percentage bias (*top*) and power (*bottom*) against correlation patient risk and refusal probability using recruitment with refusal and negative correlation between refusal and risk: both plots are paneled by the refusal probabilities and present results for all four analysis methods. The *black* reference lines represent empirical power and zero bias

Figure 4 presents the bias of the effect estimate of increasing refusal probabilities and positive correlations under recruitment with refusal. The biases are consistent with those under recruitment without refusal, with some slight variation between the two due to random variation in the sample. However, the main effect of recruitment with refusal compared to recruitment without refusal is, understandably, an increase in statistical power. The 2SRI analyses resulted in overestimation of the effect by between 0.06 and 13.5% and a power which fluctuated between 0.81 and 0.90. Figure 5 shows the effects on bias and power when increasing refusal probabilities and strengthening negative correlations under recruitment with refusal. Again, the biases follow consistent patterns across both recruitment methods. The ITT and IV analyses were above the desired statistical power for all scenarios. Of all approaches the 2SPS performed best in terms of minimum absolute bias, between −4.9 and 11.7%.

Scenarios with both clinician refusal and patient refusal greater than zero were simulated, with IV methods again providing the least biased effect estimates. The bootstrapped standard Fs were calculated to estimate precision of the effect estimate. For both IV analyses standard errors were greater than the ITT and PP analyses in all scenarios, with little difference between the standard errors of 2SPS and 2SRI. Considering refusal at only the patient level, as seen in Figs. 2, 3, 4, 5, recruitment without refusal produced standard errors of up to 0.11 under ITT analysis and 0.16 under 2SPS and 2SRI analyses. Recruitment with refusal produced consistent standard errors regardless of the correlation of around 0.08 under ITT analysis and 0.11 under 2SPS and 2SRI analyses.

## Discussion

ITT and PP analysis are equivalent and unbiased with perfect compliance and IV analysis not necessary. When refusal is present, the intervention arm comprises both individuals on intervention and individuals on standard of care. When this refusal is random and uncorrelated with event risk, ITT estimates were biased through dilution bias, bias increasing as refusal rates increased. The makeup of the event risks of the individuals in the intervention arm depends upon the direction and strength of the correlation between refusal and event risk thus inducing bias in the effect estimates. Therefore, the bias in effect estimate when using ITT analysis is also affected by this correlation between refusal and risk, small refusal rates resulted in large bias and reduction in power if correlation was high.

IV analyses provided more accurate effect estimates when refusal was correlated to event risk. Effect estimates with the smallest absolute bias of all methods were given by the 2SRI method when correlation between refusal and risk was positive and the 2SPS method when correlation between refusal and risk was negative. However, when 2SPS is less biased, it is only by a small amount, and when its more biased it is by a large amount. The 2SPS estimate was also highly effected by direction of correlation, whereas, 2SRI was relatively similar irrespective of correlation direction. The standard errors of IV estimates are larger than ITT or PP due to the two stage modelling procedure.

Sample size ignoring refusal did not provide the desired power for all methods and all scenarios, except the PP analysis and positive correlation. The large decrease or increase in statistical power under PP analyses as correlations strengthened was actually indicative of the large bias present in the effect estimate. Sample size acknowledging refusal maintained statistical power for all scenarios when using the 2SRI method and relatively small bias, only greater than 10% when correlation between risk and refusal was high.

The simulation results from this study have relevance to clinical research. Since the development of the cmRCT design uptake has been increasing in conjunction with the movement towards developing more pragmatic trial approaches in order to tackle some of the current challenges present in the clinical trial model [1]. As new cmRCT cohorts continue to emerge it is crucial that the design and analysis of these novel trials remains rigorous. Refusal of an intervention is pragmatic in the sense that that there will likely be some level of refusal to treatment in clinical practice. A certain proportion of the refusal present in a cmRCT trial will be present in real life, some individuals would refuse said treatment whether it was offered as part of a trial or not. However, refusal may be related to the fact that the intervention is offered as part of a trial. It is expected that the effect size of interest, or the effect we would observe in clinical practice, would be somewhere between that estimated with ITT and IV analyses. All present cmRCT planned ITT as the main analysis method; future research would benefit from using actual refusal rates seen in these trials and thus considering if the refusal rates used in these simulations are realistic.

It is expected that there will be patients who do not refuse the intervention, but are noncompliant to some degree. If the non-compliance present in a trial is also expected in clinical practice, we believe the intervention effect estimate can just ignore it, it is realistic of clinical practice. In a cmRCT, given the passive follow-up of the control arm, it would not make sense to use any compliance increasing tactics for the intervention arm, and its likely it would be hard to assess compliance using the routinely collected data, so lack of compliance should ideally be treated as part of treatment effect.

A key limitation of this study is that heterogeneous treatment effects were not considered and such results are not generalizable to studies with a heterogeneous treatment effect. Secondly, adding time dependent covariates to the risks may provide a more realistic scenario of how individual risks change over time. Further work could consider more complex trial scenarios that may occur in the cmRCT design. For example, when the new treatment or control treatment is harmful in terms of non-CVD mortality and how this competing risk affects the effect estimate and power of the trial mortality. The effect of multiple trials within the same cohort, which is an interesting design feature of the cmRCT, could also be investigated to assess how possible correlation of control arm affects hypothesis testing and error rates [25]. Due to the use of a cohort in a cmRCT for recruitment it would also be of interest to investigate more closely the effect of refusal on power. For example, considering a trial simulation with a fixed cohort of size n and different refusal rates, this could be used to provide an idea of scenarios when it may be more powerful to do a standard two arm trial than a cmRCT and thus preferable. As previously discussed if the cohort of eligible patients in a cmRCT is too small and the refusal high, there is the risk of oversampling the control arm leading to a less powerful trial than the standard two arm RCT.

## Conclusion

We have presented simulation results that show both the biases and statistical power of a cmRCT design are altered through refusal, particularly non-random refusal. We recommend a cmRCT design to assess probable refusal rates at the outset as well as the effect estimate, and to incorporate these into power calculations. We recommend using adaptive power calculations, updating them as refusal rates are collected in the trial recruitment phase. The instrumental variable methods provide a less biased effect estimate when refusal is present in the intervention arm. On average the 2SRI method provides effect estimates with the smallest absolute bias of all methods and sufficient power when using recruitment with refusal. We recommend running both an ITT and 2SRI analyses as it is expected that the effect size of interest, or the effect we would observe in clinical practice, would lie somewhere between those estimated with ITT and IV analyses. We demonstrate that sample sizes should be adapted based on expected and actual refusal rates in order to be sufficiently powered for instrumental variable analysis.

## Abbreviations

2SPS: Two stage predictor substitution
2SRI: Two stage residual inclusion
cmRCT: Cohort multiple randomised control trial
CVD: Cardiovascular disease
ITT: Intention-to-treat
IV: Instrumental variable
PP: Per-protocol
RCT: Randomised controlled trial
TwiCs: Trials within cohorts
