# Erratum to: Evaluation of biases present in the cohort multiple randomised controlled trial design: a simulation study

**DOI:** 10.1186/s12874-017-0326-4

**Published:** 2017-03-22

**Authors:** Jane Candlish, Alexander Pate, Matthew Sperrin, Tjeerd van Staa, on behalf of GetReal Work Package 2

**Affiliations:** 1 Health eResearch Centre, Farr Institute for Health Informatics Research, University of Manchester, Vaughan House, Portsmouth Road, Manchester, M13 9PL UK; 2 School of Health and Related Research, University of Sheffield, 30 Regent St, Sheffield, S1 4DA UK; 3 Utrecht Institute for Pharmaceutical Sciences, Utrecht University, Utrecht, The Netherlands

## Erratum

After publication of the original article [1], it came to the authors’ attention that there was an error affecting the References. The published Reference 14 [2] is incorrect, and should have cited a different article by Pate et al. [3].

Pate et al. [3] should have been included in the Reference list as 14, and the subsequent references (and citations within the article text) should have been renumbered.
